# Supplementary material for: Identification of photoperiod-induced specific miRNAs in the adrenal glands of Sunite sheep (Ovis aries)
Source: Front Vet Sci. 2022 Jul 22;9:888207. doi: 10.3389/fvets.2022.888207 (PMC9354845; doi:10.3389/fvets.2022.888207)
Supplement: Supplementary file 1 [file Data_Sheet_1.ZIP › Supplementary file/miRDeep2 file.zip!/miRDeep2 file/Know_Structure/SP42A2/oar-mir-199a.pdf]

miRBase precursor : oar-mir-199a
Total read count : 148047
oar-miR-199a-3p read count : 101409
remaining reads : 46638

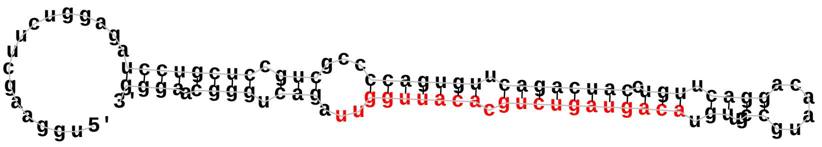

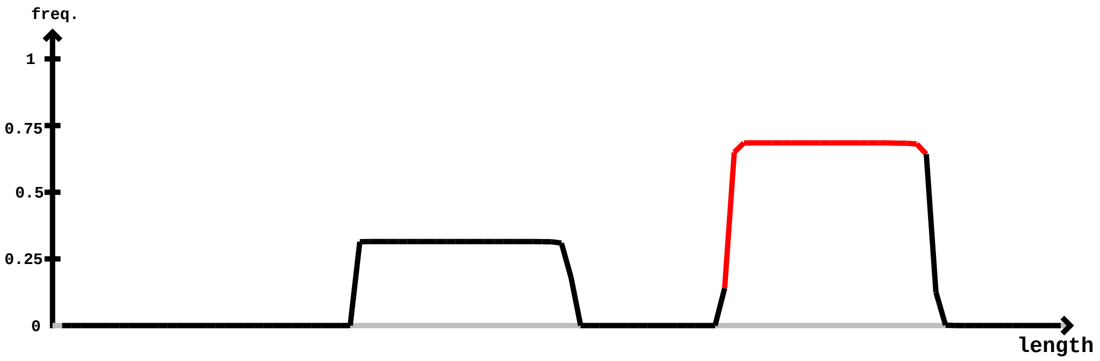

oar-miR-199a-3p

5'-	uggaagcuucuggagauccugcuccgucgccccaguguucagacuaccuguucaggacaaugccguuguacaguagucugcacauugguuagacugggcaaggg	-3'	exp	
	.................(((((((.(((...(((((((.((((((((.(((.((((......))..)).))))))))))).)))))))...))).)))).))).	reads	mm	sample
	.............................ccccaguguucagacuaccugu...	2	0	seq
	.............................ccccaguguucagacuaccuguu..	3	0	seq
	.............................ccccaguguucagacuaccuguuc...	3	0	seq
	..............................cccaguguucagacuacc..	5	0	seq
	..............................cccaguguucagacuaccu...	52	0	seq
	..............................cccaguguucagacuaccug..	68	0	seq
	..............................cccaguguucagacuaccugu...	761	0	seq
	..............................cccaguguucagacuaccuguu..	18951	0	seq
	..............................cccaguguucagacuaccuguuc...	26667	0	seq
	...............................ccaguguucagacuaccugu...	4	0	seq
	...............................ccaguguucagacuaccuguu..	41	0	seq
	...............................ccaguguucagacuaccuguuc...	73	0	seq
	................................caguguucagacuaccuguu..	2	0	seq
	................................caguguucagacuaccuguuc...	3	0	seq
	.................................aguguucagacuaccuguuc...	1	0	seq
	....aggacaaugccguuguacaguagucugcacauu..................	1	0	seq
	....aggacaaugccguuguacaguagucugcacauugguua.............	1	0	seq
	....uacaguagucugcacauu..................	51	0	seq
	....uacaguagucugcacauug.................	17	0	seq
	....uacaguagucugcacauugg................	165	0	seq
	....uacaguagucugcacauuggu...............	2595	0	seq
	....uacaguagucugcacauugguu..............	16756	0	seq
	....uacaguagucugcacauugguua.............	1191	0	seq
	....uacaguagucugcacauugguuag............	14	0	seq
	....uacaguagucugcacauugguuaga...........	1	0	seq
	....acaCuagucugcacauu..................	1	1	seq
	....acaguagucugcacauu..................	114	0	seq
	....acaguagucugcacauug.................	26	0	seq
	....acaguagucugcacauugg................	195	0	seq
	....acaguagucugcacauuggu...............	2812	0	seq
	....acaguagucugcacauugguu..............	56087	0	seq
	....acaguagucugcacauugguua.............	15968	0	seq
	....acaguagucugcacauugguuag............	164	0	seq
	....acaguagucugcacauugguuagG...........	1	1	seq
	....acaguagucugcacauugguuaga...........	11	0	seq

oar-miR-199a-3p

```
uggaagcuucuggagauccugcuccgucgccccaguguucagacuaccuguucaggacaaugccguuguacaguagucugcacauugguuagacugggcaaggg
......................................................................caguagucugcacauugg................   16     0     seq
......................................................................caguagucugcacauuggu...............   250    0     seq
......................................................................caguagucugcacauugguu..............   3738   0     seq
......................................................................caguagucugcacauugguua.............   1183   0     seq
......................................................................caguagucugcacauugguuag............   27     0     seq
......................................................................caguagucugcacauugguuaga...........   2      0     seq
.......................................................................aguagucugcacauuggu...............   1      0     seq
.......................................................................aguagucugcacauugguu..............   15     0     seq
.......................................................................aguagucugcacauugguua.............   6      0     seq
.......................................................................aguagucugcacauugguuag............   1      0     seq
.......................................................................aguagucugcacauugguuaga...........   1      0     seq
```
